# Neurofilament light chain proteins are a sensitive biomarker of neuronal damage in cirrhotic patients with hepatic encephalopathy

**DOI:** 10.3389/fmolb.2026.1744194

**Published:** 2026-02-26

**Authors:** Clelia Asero, Francesca Polito, Maria Stella Franzé, Antonio Battaglia, Claudia Ligresti, Teresa Maltese, Irene Cacciola, M’Hammed Aguennouz, Vincenzo Macaione

**Affiliations:** 1 Department of Clinical and Experimental Medicine, University of Messina, Messina, Italy; 2 Division of Medicine and Hepatology, University Hospital of Messina, Messina, Italy; 3 Section of Gastroenterology and Hepatology, PROMISE, University of Palermo, Palermo, Italy

**Keywords:** biomarkers, hepatic encephalopathy, liver cirrhosis, neurofilament light chain proteins, portal hypertension, sarcopenia

## Abstract

**Background and Aims:**

Hepatic encephalopathy (HE) is a severe complication of liver cirrhosis. Although traditionally considered reversible, growing evidence suggests that HE episodes may lead to persistent neuronal damage. This study aimed to evaluate the potential role of neurofilament light chain proteins (NfL) as biomarkers for detecting neuronal injury associated with HE.

**Method:**

Between 1 May 2024, and 30 April 2025, 133 patients were consecutively enrolled at the Liver Unit of the University Hospital of Messina. Exclusion criteria were neurological disorders and active alcohol use. Serum NfL concentrations were measured using SIMOA technology. The study design included two phases: 1) evaluation of NfL concentration differences across the study population, 2) analysis of correlations between NfL levels and clinical/biochemical parameters routinely used for HE diagnosis [West Haven criteria, ammonia levels, Animal Naming Test (ANT)], as well as anthropometric and nutritional indicators (BMI, handgrip strength, calf circumference).

**Results:**

The study population (56.4% males; median age 69 years) included 28 non-cirrhotic (21.1%) and 105 cirrhotic patients (78.9%), 34 of them (32.3%) with a diagnosis of HE. Statistical analysis revealed significant differences in NfL levels across the three groups (p = 0.001). ROC curve analysis demonstrated good discriminative ability of NfL for HE detection (p = 0.001, Area Under the Curve = 0.753). Significant correlations were observed between NfL levels and ANT scores (p = 0.001), West Haven grade (p = 0.05), ammonia levels (p = 0.021), low BMI (p = 0.009), reduced handgrip strength (p = 0.008) and calf circumference <31 cm (p = 0.043).

**Conclusion:**

NfL is a promising biomarker for HE. Its use in clinical practice may improve early detection and stratification of HE severity in cirrhotic patients.

## 1 Introduction

Hepatic encephalopathy (HE) is a frequent and serious complication of both acute and chronic liver disease. It is defined as a neuropsychiatric disorder with a broad spectrum of clinical manifestations, ranging from subtle cognitive deficits to coma (Montagnese et al., 2022). According to current international guidelines, HE is classified as covert when patients show minimal alterations detectable only through neuropsychological testing, and overt when at least temporal disorientation is present, as defined by the West Haven criteria (American Association for the Study of Liver Diseases and European Association for the Study of the Liver, 2014). The diagnosis of HE is primarily based on clinical evaluation and medical history, although plasma ammonia levels remain a key biochemical marker. Notably, low ammonia concentrations may challenge the diagnosis of HE (Rudler et al., 2021).

Although HE has traditionally been described as a reversible condition, emerging evidence suggests that cognitive impairments may persist after the clinical resolution of acute HE episodes, as well as following liver transplantation (Ochoa-Sanchez et al., 2021). This indicates that irreversible neuronal alterations may occur in patients with cirrhosis (Rose et al., 2020). Both *in vivo* and *in vitro* studies have demonstrated that HE is associated with multiple disruptions in the metabolic and energetic pathways of the neuro-glio-vascular unit (NGVU) (Claeys et al., 2021; Angelova et al., 2022).

Indeed, liver cirrhosis is characterized by elevated ammonia levels due to impaired hepatic urea cycle function and reduced extrahepatic clearance, often linked to sarcopenia (Jindal and Jagdish, 2019). Moreover, increased permeability of the blood-brain barrier, especially in the presence of portosystemic shunts, facilitates ammonia diffusion into the brain, leading to astrocytes senescence and swelling–considered irreversible–and neuronal apoptosis (Tamnanloo et al., 2023). However, ammonia alone does not fully explain the pathophysiology of HE. Clinical observation reveal discrepancies between ammonia levels and neurological symptoms, with some patients exhibiting high ammonia without overt HE and other showing HE despite low levels, suggesting the involvement of additional mechanisms (Cordova-Gallardo et al., 2025). In this context, chronic systemic inflammation appears to contribute to neuronal glucose deprivation, the onset of cerebral oedema associated with lactic acid accumulation, and the increased production of neurotoxins and radical oxygen species (Gazda et al., 2021). These alterations enhance the GABAergic tone typical of HE and promote microglial activation. This synergistic activity leads to a disruption of the central nervous system microenvironment, the development of a gliopathy, and a secondary neuronal injury–a process known as inflammaging–which may underlie the irreversible changes observed in HE (Görg et al., 2018).

In this context, the identification of novel biomarkers reflecting NGVU damage may offer valuable insights into the pathophysiology of HE. Numerous blood-based biomarkers reflecting systemic or neuronal inflammation have been investigated for HE diagnosis. Among these, several interleukins (IL) – such as IL-6, IL-17, IL-18, and IL-21 – as well as STAT3, tumour necrosis factor-α, and chemokines including CCL20, CXCL13, and CX3CL1, have been independently associated with minimal HE, highlighting the role of a pro-inflammatory environment in the onset of the disease. Biomarkers of systemic inflammation also include glutathione, malondialdehyde, and 3-nitrotyrosine, which are well-established indicators of oxidative stress related to neuronal dysfunction, as well as nitric oxide, a mediator of nitrosative stress that contributes to neuronal damage in HE through impairment of cyclic guanosine monophosphate signalling. Among markers specific of neuronal injury and inflammation related to HE, ammonia reflects microglial dysfunction, S100-β protein indicates increased blood-brain barrier permeability, and glial fibrillary acidic protein (GFAP) has been associated with astrocytic damage (Gallego et al., 2025a). Moreover, recent evidence suggests that neurofilaments light chain proteins (NfL), are sensitive indicators of neuronal injury across a wide range of neurological disorders, including both neurodegenerative and inflammatory conditions (Gallego et al., 2025b). NfL are neuronal cytoplasmatic proteins that play a crucial role in maintaining the structural integrity of the axonal cytoskeleton, and their concentrations in blood and cerebrospinal fluid have been shown to correlate directly with the degree of neuronal damage (Fiorillo et al., 2023).

The present study aims to explore the potential role of NfL as a diagnostic biomarker for HE, comparing its performance with plasma ammonia levels and other established clinical markers. The ultimate goal is to evaluate its applicability in routine clinical practice for the detection and stratification of HE.

## 2 Materials and methods

### 2.1 Inclusion criteria and data collection

All patients attending the Liver Unit of the University Hospital of Messina between 1 May 2024, and 30 April 2025, who provided written informed consent, were prospectively enrolled. Patients were excluded if they had a diagnosis of alcohol use disorder (AUD) or reported any alcohol consumption within the 12 months preceding the enrolment (European Association for the Study of the Liver, 2018). Additional exclusion criteria included illicit drug use and the presence of neurological conditions that could potentially confound the assessment of cognitive function. These included stroke, transient ischemic attack (TIA), chronic cerebral vasculopathy, Parkison’ disease, peripheral neuropathy, Alzheimer disease, dementia, type 2 diabetes neuropathy, and traumatic brain injury. Participants were categorized into three cohorts: *(1)* patients with liver cirrhosis and a confirmed diagnosis of HE, *(2)* patient with liver cirrhosis without HE, and *(3)* non-cirrhotic patients, who served as negative controls.

At the time of enrolment, comprehensive data was collected for each participant, including demographic and anthropometric characteristics, clinical history, instrumental findings, and biochemical parameters. All information was systematically recorded in a digitized database to ensure accuracy and facilitate subsequent analysis. Liver cirrhosis was diagnosed based on a combination of clinical findings, ultrasonographic imaging, and elastographic measurements, in accordance with established criteria. The severity of liver cirrhosis was assessed using the Child-Pugh scoring system, which stratifies patients into three classes–A, B, or C–based on clinical and biochemical parameters. At the time of enrolment, liver decompensation was systematically assessed in accordance with international guidelines. Specific clinical features considered included the presence of ascites, episodes of gastrointestinal bleeding, and manifestations of HE, all of which reflect impaired hepatic function and disease progression (Angeli et al., 2018).

The diagnosis of HE was established through a structured clinical evaluation, which included the assessment of flapping tremor (asterixis), performing of Animal Naming Test (ANT), and evaluation of the patients’ orientation in time and space. These clinical findings were complemented by the measurement of plasma ammonia levels, providing biochemical support for the diagnosis (Montagnese et al., 2022).

Demographic data collected for each participant included age and sex. Anthropometric assessment comprised measurements of body mass index (BMI), calf circumference, and handgrip strength. Calf circumference was measured at the widest point of the lower leg, and values below 31 cm were considered indicative of reduced muscle mass and classified as pathological. Handgrip strength test was evaluated using a Jamar dynamometer, following standardized protocols. Thresholds for reduced strength were <27 kg in men and < 16 kg in women (Cruz-Jentof et al., 2019).

Biochemical parameters included serum values of aspartate aminotransferase (AST), alanine aminotransferase (ALT), glutamyl transpeptidase (GGT), alkaline phosphatase (ALP), lactic dehydrogenase (LDH), platelets (PLT), prothrombin time (PT), albumin, gamma-globulins, total bilirubin, creatinine, sodium, total cholesterol, fasting glucose, and glycated hemoglobin (HbA1c). Liver stiffness measurement (LSM) was performed using the FibroScan instrument with either M or XL probes, according to the manufacture indications. Ultrasound was used to assess portal vein diameter and spleen interpolar diameter, while abdominal computed tomography (CT) and magnetic resonance imaging (MRI) were employed to detect portosystemic shunts. Cirrhotic patients also underwent upper gastrointestinal endoscopy for the evaluation of esophageal varices, according to Baveno VII recommendations (de Franchis et al., 2022). The study design comprised two distinct phases. In the first phase, we assessed the distribution and variability of NfL concentrations across the entire patient cohort, aiming to identify potential differences linked to HE. The second phase focused on exploring the relationship between NfL levels and a range of clinical and biochemical markers routinely employed in the diagnosis and staging of HE, including the West Haven criteria, plasma ammonia concentrations, and the ANT. Additionally, we investigated correlations with anthropometric and nutritional indicators–such as BMI, handgrip strength, and calf circumference–to evaluate the potential influence of nutritional status and muscle function on NfL levels.

The study was conducted in accordance with the ethical standards of the institutional research committee and the Declaration of Helsinki. The study protocol was approved by the Institutional Review Board of district of Messina (protocol number Prot. 90–23 del 10/05/2023).

### 2.2 Molecular analyses

Blood samples for NfL detection were collected from all patients, centrifuged within 1 hour of collection, aliquoted in polypropylene tubes and stored at −80 °C until molecular analyses. Single Molecule Array technology (SIMOA, Quanterix) was used to measure NfL, as it identifies and quantifies immunocomplexes bound to dye-encoding magnetic beads (using different capture and detector antibodies) sealed in arrays of femtoliter-volume microwells. Rapid‐thawed plasma was centrifuged at 10,000xg for 10 min, 25 μL (diluted 4‐fold in buffer) was added to kit beads (100 μL) by pipette in each well, the plate was incubated for 15 min at 30 °C, magnetic‐washed 3x for 5 min total, subjected to addition of SBG reagent (100 μL), followed by another incubation for 10 min at 30 °C, washed again x five for 7 min total, and reading on the SIMOA SR‐X machine. All measurements were duplicated to assure inter-assay precision by calculating the coefficient of variation (CV). Results with CVs below 20% were accepted for statistical analysis. The reference values for age groups are as follows: 40–60 years: <17 pg/mL; 60–75 years: <28 pg/mL; >75 years: <35 pg/mL (Alberti et al., 2025; Vermunt et al., 2022).

### 2.3 Statistical analysis

Numerical data were expressed as median and interquartile range (IQR, first and third quartile), whereas categorical variables were presented as number and percentage. The non-parametric approach was adopted because most numerical variables were not normally distributed, such as those verified by Kolmogorov–Smirnov test. The Kruskal–Wallis Test was performed to compare neurofilaments levels among the three groups of patients: non-cirrhotic, cirrhotic with HE, and cirrhotic without HE. Bonferroni’s *post hoc* correction was used to identify which groups significantly differed from each other. Subsequently, the Mann-Whitney U Test was used to compare NfL levels between patients with and without HE, as well as to assess differences according to the presence of collateral vessels and esophageal varices. The Spearman’s Rho correlation Test was employed to evaluate significant associations between NfL levels and the scores of the ANT, West Haven grades and ammonia levels. Mann Whitney U test and Fisher’s exact test were also be applied to evaluate the features of the patients with pathological levels of NfL. Receiver Operating Characteristic (ROC) curve analysis was used to assess the ability of neurofilament levels to discriminate between non-cirrhotic patients with hepatic encephalopathy, reporting the area under the curve (AUC) with 95% confidence interval.

## 3 Results

### 3.1 Study population descriptive analysis

A total of 133 patients consecutively attending the Liver Unit of the University Hospital of Messina from 1 May 2024, to 30 April 2025, who met the inclusion criteria, were enrolled in the study. The cohort comprised 75 males (56.4%) and 58 females (43.6%), with a median age of 69 years (range 60–76). Overall, 28 patients (21.1%) were classified as non-cirrhotic, while 105 (78.9%) had a diagnosis of liver cirrhosis. Among the cirrhotic patients, 34 individuals (32.4%) had a documented history of HE, and 30 of these (88.2%) were receiving targeted pharmacological treatment for HE at the time of enrolment.

Regarding the severity of cirrhosis, 71 patients (53.38%) were classified as Child Pugh A, 29 (21.8%) as Child Pugh B, and 5 (3.8%) as Child Pugh C. The predominant etiology of liver disease was MASLD, affecting 61 patients (45.9%), followed by chronic viral infections in 34 patients (25.8%). At the time of enrolment, 39 patients (29.3%) had portosystemic shunts and 59 (44.4%) had esophageal varices. Thirty-seven patients presented signs of liver decompensation, including 29 (21.8%) with ascites and 8 (6%) with gastrointestinal bleeding. Furthermore, 60 (45.1%) had a history of prior hepatic decompensation, and 5 (3.8%) had undergone Transjugular Intrahepatic Portosystemic Shunt (TIPS) placement. Flapping tremor, a clinical sign indicative of HE, was observed in 15 patients (11.3%) during clinical evaluation. The median ANT score was 15 (range 11–15), reflecting mild cognitive impairment in patients with HE. The median NfL concentration was 49.65 pg/mL (range 11.48–31.80 pg/mL), while the median ammonia concentration was 64.9 μmol/L (range 41.72–87.75 μmol/L). According to the West Haven criteria, 12 patients (9%) were classified as grade I HE, and six patients (4.5%) were classified as grade II HE. The median calf circumference was 36 cm (range 34–38 cm), and the median handgrip strength was 29 kg (range 23.7–40.5 kg). Notably, 10 patients (7.5%) exhibited signs suggestive of sarcopenia, defined by reduced handgrip strength, in accordance with established diagnostic criteria. Comparative analysis among the three groups revealed no significant differences in sex distribution (p = 0.475) or anthropometric parameters including BMI (p = 0.593), calf circumference (p = 0.145) and handgrip strength test (p = 0.241) indicating a homogeneity of the study population. Conversely, patients in the three cohorts differed for age, biochemical and instrumental variables associated to liver disease severity (p < 0.001) as indicated in Table 1.

**TABLE 1:** Demographic, clinical, and biochemical features of 133 patients included in the study. Numerical variables are expressed as median (first – third quartile), and categorial variables as absolute number (percentage).

Features	Patients (n. 133)	Cirrhotic with HE (n.34)	Cirrhotic without HE (n.71)	Non-cirrhotic (n.28)	P value
Male sex, n Female sex, n	75 (56.4) 58 (43.6)	22 (64.7) 12 (35.3)	37 (52.1) 34 (47.9)	16 (57.1) 12 (42.9)	0.475
Age, years	69 (60–76)	71 (63–77)	72 (63–77)	59 (45–63)	**< 0.001**
BMI, kg/m^2^	27.66 (24.27–30.42)	28.32 (23.69–32.48)	27.26 (23.55–30.23)	27.52 (25.06–30.24)	0.593
Calf circumference, cm	36 (34–38)	35 (30.5–37)	36 (34–38.25)	38.5 (34.75–45.25)	0.145
Handgrip strength test, kg	29 (23.7–40.5)	28 (23.7–36)	29 (23–43)	42.5 (28.25–51.5)	0.241
Duration of liver disease, years	5 (2–10)	6 (2–11)	5 (2–10)	3 (1–6)	0.083
Liver stiffness, kPa	17.75 (7.9–28.47)	31.6 (15.9–42.2)	19.9 (13.8–28.25)	6.25 (4.7–7.52)	**< 0.001**
ANT, number in 60″	15 (11–15)	11 (7–15)	15 (11–15)	15 (15–15)	**< 0.001**
Ammonia, µmol/L	64.9 (41.72–87.75)	83 (63.6–144)	58.45 (40.95–81.2)	45.8 (22.25–74.5)	**0.001**
AST, U/L	32.95 (25.17–41.12)	34.15 (27.5–51)	32 (26.4–40.3)	29 (21–34)	0.085
ALT, U/L	27 (20–36.75)	22.5 (18–37)	26 (20.2–35)	33 (20–62)	**0.047**
GGT, U/L	52.5 (27.25–96.75)	47 (26.5–112.75)	60 (28–98)	43 (25–111)	0.873
Total bilirubin, mg/dL	1 (0.677–1.53)	1.35 (0.75–2.41)	1.06 (0.7–1.4)	0.7 (0.5–1.6)	**0.004**
PLT/mm^3^	117 (75–191)	82 (58–116)	116 (76–151)	243 (199–263)	**< 0.001**
PT	1.15 (1.06–1.28)	1.25 (1.13–1.44)	1.15 (1.09–1.27)	1.01 (0.99–1.12)	**< 0.001**
Albumin, g/dL	3.8 (3.34–4.25)	3.27 (2.81–3.66)	3.8 (3.46–4.12)	4.3 (3.92–4.54)	**< 0.001**
Gamma-globulins, g/dL	1.29 (1.03–1.67)	1.5 (1.15–1.77)	1.39 (1.02–1.7)	1.13 (0.91–1.19)	**0.006**
Total cholesterol, mg/dL	137.5 (116.5–170)	131 (92–148)	132 (115 - 165)	148 (175–193)	**< 0.001**
Creatinine, mg/dL	0.9 (0.73–1.18)	0.92 (0.73–1.34)	0.9 (0.71–1.18)	0.90 (0.73–1.05)	0.707
NfL, pg/mL	19.65 (11.48–31.8)	25.32 (15.35–47.58)	20.7 (12.35–31.57)	11.98 (8.93–18.62)	**0.001**
Esophageal varices, n	59 (44.4)	22 (64.7)	37 (52.1)	0 (0)	**< 0.001**
Gastrointestinal bleeding, n	8 (6)	4 (11.8)	4 (5.6)	0 (0)	0.150
Ascites, n	29 (21.8)	15 (44.1)	14 (19.7)	0 (0)	**< 0.001**
Previous liver decompensation, n	60 (45.1)	29 (85.3)	31 (43.7)	0 (0)	**< 0.001**
NSBB, n	67 (50.4)	20 (58.8)	43 (60.6)	4 (14.3)	**< 0.001**
Portal vein diameter, mm	14 (12–16)	14 (12–17.5)	15 (12–16)	11 (11–12)	**0.006**

Statistically significant p-values are highlighted in bold.

Abbreviations: BMI, body mass index; ANT, animal naming test; HE, hepatic encephalopathy; AST, aspartate aminotransferase; ALT, alanine aminotransferase; GGT, gamma-glutamyl transferase; PLT, platelets; PT, prothrombin time; NfL, neurofilament light chain proteins; NSBB, non-selective beta blockers.

### 3.2 Application of NfL for HE detection

Non-parametric analysis using the Kruskal–Wallis Test revealed statistically significant differences in NfL levels among the three study groups (H = 14.665, gl = 2, p = 0.001). Mean rank analysis showed a progressive increase in NfL levels from non-cirrhotic patients (mean rank = 43.13) to cirrhotic patients with HE (mean rank = 78.94). Bonferroni’s *post hoc* correction identified significant differences between.

- non-cirrhotic patients (mean rank 34.73) and cirrhotic patients without HE (mean rank 56.02), with higher NfL levels in cirrhotic patients (U = 566.500, Z = −3.321, p = 0.001);
- non cirrhotic patients (mean rank = 22.89) and cirrhotic patients with HE (mean rank = 38.59, U = 235.000, Z = −3.409, p = 0.001).

No significant differences were observed between cirrhotic patients with (mean rank = 57.85) and without HE (mean rank = 50.68, U = 1042.000, Z = −1.130, p = 0.259).

**FIGURE 1:**
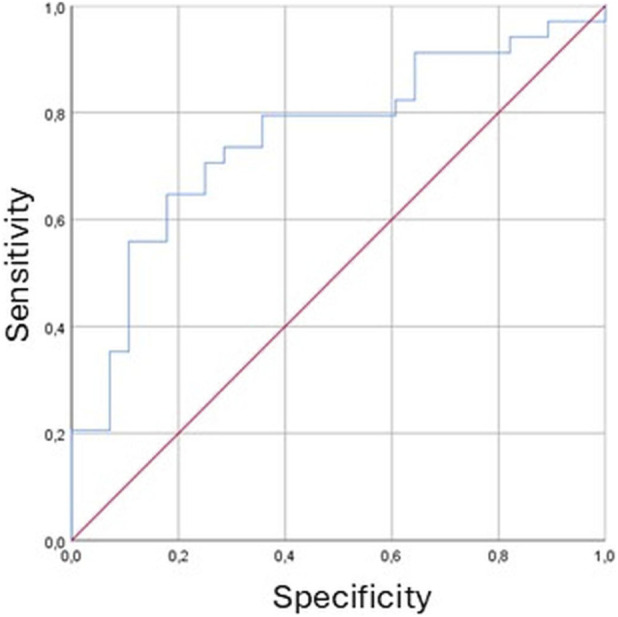
Receiver Operating Characteristic (ROC) curve of neurofilament light chain proteins (NfL) levels for the discrimination between patients with hepatic encephalopathy (HE) and non-cirrhotic patients. The area under the curve (AUC) was 0.753 (95% C.I. 0.630–0.876; p = 0.001), indicating good discriminative performance of the test. Figure Legend: Red diagonal line represents reference line with AUC = 0.5. The x-axis represents the specificity of the test, while the y-axis represents the sensitivity.

ROC curve analysis was performed to evaluate the ability of NfL levels to discriminate between patients with HE and non-cirrhotic patients. The analysis demonstrated good discriminative performance, with an AUC of 0.753 (95% C.I. 0.630–0.876, p = 0.001) (Figure 1).

**FIGURE 2:**
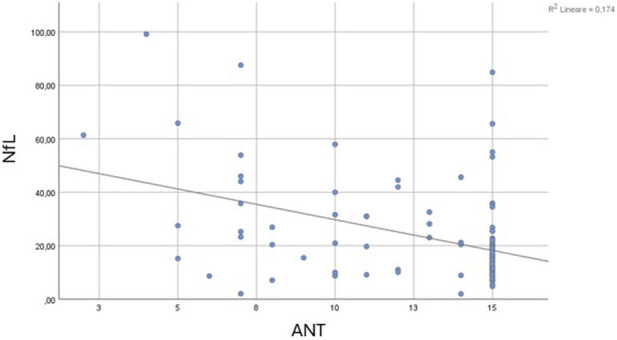
Scatter plot showing the relationship between serum neurofilament light chain (NfL, pg/mL) concentrations and Animal Naming Test (ANT) scores. A moderate negative correlation was observed (Spearman’s Rho = −0.320, p = 0.001), indicating that higher NfL levels were associated with poorer cognitive performance (R^2^ = 0.174).

**FIGURE 3:**
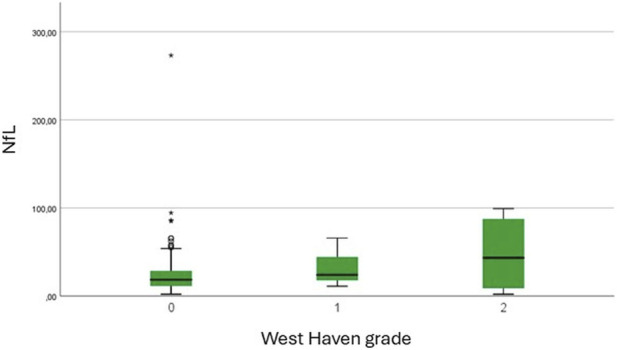
Boxplot shows serum neurofilament light chain (NfL, pg/mL) concentrations according to West Haven grade. Median NfL levels progressively increased with higher hepatic encephalopathy grades, consistent with a weak but significant positive correlation (Spearman’s Rho = 0.168, p = 0.05). Outliers are indicated by circles (moderate) and asterisks (extreme).

**FIGURE 4:**
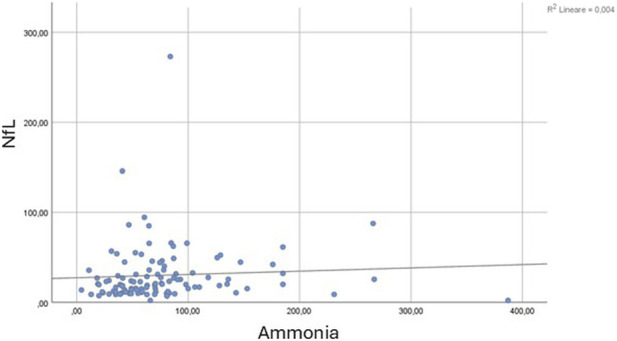
Scatter plot showing the relationship between serum neurofilament light chain (NfL, pg/mL) and ammonia levels (µmol/L). A weak positive correlation was observed between NfL and ammonia concentrations (Spearman’s Rho = 0.222, p = 0.021), with a minimal proportion of NfL variability explained by ammonia levels (R^2^ = 0.004).

The Mann-Whitney U test confirmed significantly higher NfL levels in patients with HE (mean rank = 78.94) compared to those without HE (mean rank = 62.90, U = 1277.0, Z = −2.094, p = 0.036). When comparing patients with (mean rank = 73.05) and without portosystemic collateral vessels (mean rank = 63.01), no significant differences were observed (U = 1519.000, Z = −1.384, p = 0.166). Spearman’s correlation Test revealed a moderate negative correlation between NfL levels and ANT scores (Rho = −0.320, p = 0.001), suggesting that higher neurofilaments levels were related to poorer cognitive performance (Figure 2). A low-grade positive association was found between NfL levels and West Haven grade (Rho = 0.168, p = 0.05), indicating that higher NfL concentrations corresponded to more severe HE grades (Figure 3). Ammonia levels showed a weak positive correlation with NfL concentrations (Rho = 0.222, p = 0.021, Figure 4).

### 3.3 Correlations between pathological NfL levels and clinical features

Following data collection, patients were stratified according to the presence of pathological NfL levels, based on age-adjusted reference intervals (Alberti et al., 2025; Vermunt et al., 2022). To explore potential association with clinical and biochemical parameters, both the Mann-Whitney U and the Fisher’s exact Test were applied.

Mann-Whitney U analysis revealed that patients with pathological NfL values had older age (mean rank 84.8 vs*.* 58.79, U = 1163.500, Z = −3.621, p < 0.001), and exhibited lower BMI (mean rank 51.25 vs*.* 70.09, U = 1206.500, Z = −2.626, p = 0.009) and reduced muscle strength (mean rank 25.88 vs*.* 38.39, U = 318.500, Z = −2.645, p = 0.008). They also present lower ANT scores (mean rank 37.76 vs*.* 55.36, U = 619.000, Z = −2.939, p = 0.003), indicating impaired cognitive functions. Biochemically, these patients showed higher ammonia concentrations (mean rank 65.14 vs*.* 48.96, U = 920.000, Z = −2.548, p = 0.011) and elevated MELD scores (mean rank 63.32 vs*.* 46.52, U = 839.000, Z = −2.735, p = 0.006), reflecting more advanced liver disfunction. In addition, they had significantly lower levels of serum ALT (mean rank 55.27 vs*.* 71.74, U = 1418.500, Z = −2.304, p = 0.021), albumin (mean rank 48.73 vs. 72.86, U = 1143.500, Z = −3.436, p = 0.001), and cholesterol (mean rank 50.87 vs*.* 71.16, U = 1233.500, Z = −2.906, p = 0.004), suggesting compromised liver synthetic function and poorer nutritional status. Interestingly, creatinine levels were markedly higher in patients with pathological NfL (mean rank 90.99 vs*.* 53.34, U = 777.500, Z = −5.334, p < 0.001). Fisher’s exact test further highlighted significant associations between pathological NfL and reduced calf circumference (p = 0.043), use of non-selective beta-blockers (NSBB) (p = 0.001), and presence of liver decompensation (gastrointestinal bleeding and ascites, both p = 0.012) at the enrolment or before (previous episodes of liver decompensation p = 0.001). These findings suggest that elevated NfL may be indicative of both hepatic and extrahepatic deterioration.

## 4 Discussion

Hepatic encephalopathy represents a major public health burden, being a frequent cause of hospitalization and readmission in the liver units after discharge for patients with liver cirrhosis. Its impact extends beyond the clinical setting, as it severely compromises patients’ quality of life, worsens overall prognosis, and contributes to increase mortality (Elsaid and Rustgi, 2020). Despite decades of research, the pathophysiology of HE is still not fully understood. However, emerging evidence points to a central role of NGVU dysfunction in the onset of minimal HE and persistence of overt HE-related neurological symptoms (Labenz et al., 2021; Cheng et al., 2025). In this context, our study explored the potential diagnostic value of NfL–which are well established biomarkers of neuronal injury–in the diagnosis of HE (de Wit et al., 2024).

Serum NfL concentrations were measured across the three groups of patients with different degrees of liver disease severity. The results revealed significant differences in NfL expression among these groups (p = 0.001), with notably higher concentrations observed in cirrhotic patients compared to non-cirrhotic individuals (p = 0.001), and in those with HE compared to those without (p = 0.036). The association between elevated NfL and HE was further reinforced by correlation analyses. NfL levels showed a positive correlation with blood ammonia concentrations and with HE severity as assessed by West Haven criteria (p = 0.021 and p = 0.05, respectively). A strong correlation was observed between NfL and ANT scores (p = 0.001), suggesting that higher NfL levels may reflect greater cognitive impairment in cirrhotic patients. Finally, the diagnostic performance of NfL in identifying HE was confirmed through ROC curve analysis, which yielded an area under the ROC curve (AUC) of 0.753 (p value = 0.001), indicating good discriminative ability. Altogether, these findings support the potential utility of serum NfL as a non-invasive biomarker for the detection and grading of HE in patients with liver cirrhosis. This biomarker-based approach could offer meaningful diagnostic and prognostic advantages, particularly in clinical scenarios where traditional markers such as ammonia levels fall short in capturing the complexity and reversibility of HE. Moreover, the integration of NfL into clinical practice may open new avenues for research and monitoring, especially in the context of liver transplantation and neurocognitive outcomes.

Our study contributes to a deeper understanding of the pathophysiological mechanisms underlying HE, particularly by highlighting the strong interrelationship between HE, sarcopenia, and caloric–protein malnutrition. Sarcopenia is frequently associated with liver cirrhosis, affecting an estimated 65%–90% of individuals with chronic liver disease, and is increasingly recognized as a key factor in the development and progression of HE. This connection is largely attributed to the role of skeletal muscle in extrahepatic ammonia detoxification, as muscle tissue incorporates ammonia into glutamine. This compensatory mechanism becomes significantly impaired in the presence of sarcopenia (Lattanzi et al., 2019). Moreover, the relationship between hyperammonemia and sarcopenia appears to be bidirectional. Elevated ammonia levels can exacerbate muscle wasting through the activation of myostatin, a negative regulator of muscle growth, which promotes mitochondrial dysfunction, reactive oxygen species production, and skeletal muscle proteolysis, ultimately accelerating muscle catabolism (Mendez-Guerrero et al., 2024; Marrone et al., 2023; Nardelli et al., 2019). In recognition of this interplay, international guidelines advocate for adequate dietary protein supplementation in patients with sarcopenia and HE, as nutritional support may help interrupt this vicious cycle (European Association for the Study of the Liver, 2019). In our cohort, elevated NfL concentrations were significantly associated with several indicators of poor nutritional and functional status, including low serum albumin levels (p = 0.001), calf circumference below 31 cm (p = 0.043), reduced handgrip strength (p = 0.008), and lower BMI values (p = 0.009). These findings suggest that NfL may not only reflect neuronal injury but also serve as an indirect marker of sarcopenia and malnutrition, further reinforcing the complex and multifaceted nature of HE in cirrhotic patients.

Beyond the correlation with HE, our analysis also revealed that elevated NfL were significantly correlated with features indicative of clinically relevant portal hypertension. Specifically, higher NfL concentrations were associated with the use of NSBB (p = 0.001) and the presence of hepatic decompensation (p = 0.012), both of which are established markers of advanced portal pressure and disease severity. Additional correlations emerged with laboratory indicators of progressive liver dysfunction, including higher MELD scores (p = 0.006), hypoalbuminemia (p = 0.001), and hypercholesterolemia (p = 0.004). These findings suggest that NfL may reflect not only neurological impairment, but also systemic deterioration linked to cirrhosis progression.

Significant associations were also observed with older age (p < 0.001) and elevated serum creatinine levels (p < 0.001). While the relationship between NfL levels and aging is well documented–likely due to physiological reduction in cerebrospinal fluid turnover and to age-related axonal damage–the correlation with serum creatinine remains controversial (Lu et al., 2024). Previous studies have proposed that renal function may influence circulating NfL concentration, a hypothesis supported by our data (Liu et al., 2024; Tang et al., 2022). It is plausible that, in our cohort, elevated NfL levels may reflect impaired renal clearance, either due to chronic kidney disease or the renal dysfunction commonly associate with liver cirrhosis (Jung and Chang, 2023; Low et al., 2015).

Collectively, these findings reinforce the potential of serum NfL as a sensitive biomarker for HE. Its integration into routine clinical practice–alongside traditional assessments such as ammonia levels and clinical evaluation–could enhance the early detection and stratification of HE, enabling more tailored and proactive management strategies for patients at highest risk.

This study has several limitations, the most relevant being the lack of data on serum levels of biomarkers related to systemic and neuronal inflammation other than NfL. The inclusion of these biomarkers in future studies may further strengthen the interpretation of our findings by providing additional insight into the pathophysiological mechanisms underlying HE and by supporting the development of more comprehensive, prospective investigations in this field.

## 5 Conclusion

Hepatic encephalopathy continues to pose a substantial burden on public health systems, given its high prevalence among patients with liver cirrhosis and its profound impact on quality of life, morbidity, and healthcare utilization. Early recognition and timely intervention remain critical to improving outcomes and preventing irreversible neurological damage. Our findings highlighted that NfL may serve as valuable biomarkers to identify patients at greater risk of neuronal injury and cognitive decline, particularly in presence of advanced liver disease or nutritional impairment. Ultimately, incorporating NfL into the diagnostic and monitoring framework for HE could enhance clinical decision-making, reduce hospital re-admissions, and mitigate the broader social and economic impact of this debilitating condition.

## Data Availability

The data are available upon reasonable request to the corresponding author.
